# Changing Trends in Conscious Sedation at Pediatric Dental Offices: A Bibliometric Analysis

**DOI:** 10.7759/cureus.40191

**Published:** 2023-06-09

**Authors:** Pooja V R, Victor Samuel A, Kavitha Ramar

**Affiliations:** 1 Department of Pediatric and Preventive Dentistry, SRM Kattankulathur Dental College and Hospital, Kattankulathur, IND

**Keywords:** clinical pediatric dentistry, moderate sedation, pediatric dental treatment, bibliometric analysis, conscious sedation

## Abstract

﻿The uncooperative behavior of children in the dental office has increased the demand for pharmacological behavior management techniques. Moderate sedation is more effective by providing analgesia and anxiolysis, thereby aiding in carrying out the most comfortable, efficient, and high-quality dental services. There is a need to understand the various aspects such as the choice of drug, method of drug administration, safety, and efficacy. Bibliometrics can reveal significant shifts in research and publication trends. Therefore, this study aimed to perform a bibliometric analysis of the literature on evolving trends in conscious sedation at pediatric dental offices. The bibliometric research used RStudio 2021.09.0+351 for Windows (RStudio, Boston, MA), package "bibliometrix," and VOS viewer software (Centre for Science and Technology Studies, Leiden University, The Netherlands. https://www.vosviewer.com). Elsevier's Scopus database (www.scopus.com) provided the literary data for this study, which were exported in BibTex format. The articles were independently categorized according to the following aspects: (a) annual scholarly output; (b) leading countries or regions; (c) leading journals; (d) productive authors; (e) citations; (f) study design; and (g) distribution of topics. The findings considered 1996 through 2022 and used journals, books, articles, and other sources, totaling 1064 papers, with an average of 10.7 per year. According to the findings, the United States, the United Kingdom, and India were principally leading the field of conscious sedation research. In total, 2433 authors were found through the search. The study identified the nations that are currently researching the topics such as midazolam and nitrous-oxide; this paves the way for future partnerships to strengthen the evidence generated in other topic areas using novel sedatives and exploring the different routes of drug administration, thereby benefiting the scientific community by identifying knowledge gaps and experts in this area of research.

## Introduction and background

Pediatric dentists face highly challenging situations in managing the child's behavior, an essential element of pediatric dental education. ﻿Uncooperative behavior in the dental office is frequently attributed to behavioral manifestations of anxiety. A significant population of children is exceptionally uncooperative, fearful, anxious, or physically resistant, particularly during invasive procedures and parental separation [1]. Such behavior may have serious repercussions, including delaying or terminating treatment or decreasing the quality of dental care provided [2]. Compared to contemporary techniques, today's modern pediatric dentistry describes a wide range of nonpharmacological and pharmacological behavior management approaches to effectively control the behavior of young dental patients. Nonpharmacological techniques typically reduce most children's unnecessary fear and anxiety. Still, a sizeable percentage of patients provides a significant challenge to the pediatric dentist, impeding the effective and efficient provision of vital dental care.﻿ If these techniques prove inadequate, the application of pharmacological techniques such as sedation or general anesthesia is indicated [3]. In pediatric dentistry, the main goal of pharmacological sedation is to alter the patient's behavior to a degree that enables us to deliver efficient care. The physiological consequences of sedation vary greatly depending on the medicine, dose, method of administration, and patient characteristics [4]. "Moderate sedation is defined as a medically induced, depressed consciousness state that maintains protective reflexes, keeps the patient's capacity to independently and consistently maintain a patent airway, and allows the patient to respond appropriately to physical or verbal commands" [5]. Moderate sedation is more effective by providing analgesia and anxiolysis, thereby aiding in carrying out the patient's most comfortable, efficient, and high-quality dental services and developing a positive attitude toward future dental treatment. Over the years, a plethora of sedative agents was discovered, such as nitrous oxide, opioids, chloral hydrate, ketamine, midazolam, propofol, sevoflurane, etc., each having its advantages and limitations [4].

These medications have been used individually or in combination for decades in pediatric sedation [6]. Despite their effectiveness, the associated adverse effects limit their use in pediatric dental procedures. Research in conscious sedation in pediatric dental practice is emerging in various aspects like the choice of drug, method of drug administration, and its safety and efficacy, but the quest is still ongoing. Immense qualitative research in conscious sedation has been addressed in the literature, but there is no quantitative research to date. Thus, this paper aims to handle massive scientific data and generate highly influential research by conducting a bibliometric analysis of conscious sedation in pediatric dentistry.

The objective of this bibliometric analysis is to investigate the theoretical foundation of conscious sedation in the current literary works and identify developing trends and research elements.

## Review

Bibliometric methodology

Data Source

This bibliometric analysis used the Scopus database (www.scopus.com) to conduct a thorough literature search. The keywords such as "conscious sedation," and "pediatric dentistry" were used. The authors (VR P and AV) independently scanned the title of all the articles identified through Elsevier's Scopus database. Titles were screened for relevance in pediatric dentistry. Articles involving adults, treatment under general anesthesia, premedication using sedatives, and studies exclusively on the drugs' pharmacokinetics and pharmacodynamics were eliminated. There were no restrictions applied on language or publication status.

Data Extraction

In this study, manual and software-assisted data extraction was carried out in two segments. Two investigators (VR P and AV) separately assessed the titles of included articles for manual analysis. Following this, the information about the study design, distribution of topics, types of sedatives, and routes of administration was extracted. The results were then compared by the third researcher (K R). In the event of coincidence, it was noted as the final data. All three investigators discussed the discrepancies and then came to a consensus of including them in the review.

For the software process, data were extracted in BibText format from the Scopus database, and using RStudio (biblioshiny) (RStudio, Boston, MA) and VOSviewer 1.6.7 software (Centre for Science and Technology Studies, Leiden University, The Netherlands. https://www.vosviewer.com), analyzing and visualization of bibliometric networks was done [7]. This study used two bibliometric analysis methods - the main and the enrichment methods. Performance analysis and science mapping are the main techniques used in this research. By assessing indicators linked to citations, publications, and citations and publications, performance analysis determines the contribution of research constituents. Similarly, scientific mapping evaluates the citation and co-word analyses to explore the relationships between research constituents. Finally, by applying clustering routes, enrichment techniques were used to improve the results [8].

Results

**Figure 1:**
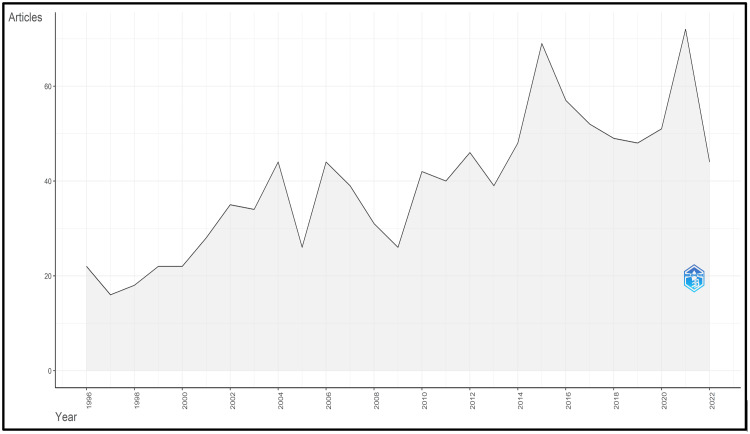
Annual scientific production

Initially, 2,147 articles were identified. Thousand and one articles were eliminated following the initial screening. Duplicate articles and papers unrelated to conscious sedation in pediatric dentistry were eliminated during the subsequent screening. Finally, 1,064 articles were included in the bibliometric study. From 1996 to 2022, 1,064 articles about conscious sedation in pediatric dentistry were published and indexed in Scopus, with an average of 10.7 per year. The number of papers on conscious sedation was generally increasing over this time. The year 2021 had the highest number of papers published (72/1,064), while 1997 had the lowest number of articles published (16/1,064) (Figure 1).

**Figure 2:**
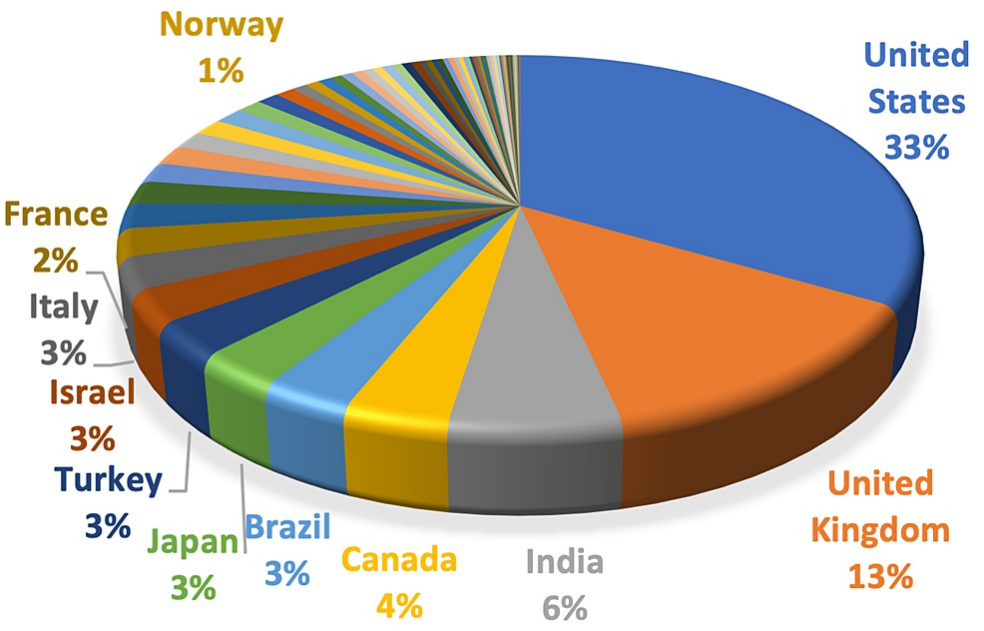
Countries' contribution

Publications on conscious sedation included contributions from 75 nations or regions. Ten countries had more than 20 publications, eight had more than 50, and two had more than 200. The top 10 countries which published 73% (776/1064) of the articles are shown in Figure 2. The most productive nation was the United States (33%, 351/1064), followed by the United Kingdom, India, Canada, Brazil, Japan, Turkey, Israel, Italy, and France. Three nations - India, Brazil, and Turkey - were developing nations, whereas the other seven were industrialized nations.

Over 25 years, the 1064 articles were published in 379 peer-reviewed publications. With a cumulative proportion of 33% in conscious sedation and 9.4% accounting for pediatric dentistry journals, the top 12 journals (351/1064), according to Bradford's law [9] met the core journals' requirements. Table 1 shows the top 10 productive journals.

**Table 1:** Top 10 journals with publications on conscious sedation

S. no.	Journal	H-Index	Publications	% of total
1.	Pediatric Dentistry	28	100	9
2.	British Dental Journal	17	45	4
3.	Journal of Clinical Pediatric Dentistry	13	39	4
4.	International Journal of Pediatric Dentistry	12	34	3
5.	European Archives of Pediatric Dentistry	12	24	2
6.	Anesthesia Progress	11	22	2
7.	Journal of Dentistry for Children	11	22	2
8.	Special Care in Dentistry	10	18	2
9.	Pediatric Anesthesia	10	16	2
10.	Current Opinion in Anaesthesiology	10	13	1

There are 2433 authors identified for the 1064 articles. Over five articles were published by 60 authors and over 10 by 13. Table 2 lists the top 10 most productive authors in conscious sedation. With 35 publications, Wilson S from Washington has published the most articles on conscious sedation, followed by Cote CJ and Girdler.

**Table 2:** Top 10 productive authors in the field of conscious sedation total citation (TC), net production (NP), and starting year (PY_start)

Element	H_index	TC	NP	PY_start
Wilson S	17	826	35	1996
Cote CJ	10	992	17	2000
Girdler NM	10	286	12	2000
Mason KP	9	366	15	2010
Costa LR	8	164	24	2012
Kupietzky A	8	170	14	1996
Ashley PF	7	186	11	2005
Costa PS	7	134	13	2012
Faulks D	7	135	7	2004
Green SM	7	1040	8	2000
Hennequin M	7	140	8	2004
Krauss B	7	1031	7	2000
Peretz B	7	198	14	1996
Welbury RR	7	246	7	1996
Wilson KE	6	182	10	2002

**Figure 3:**
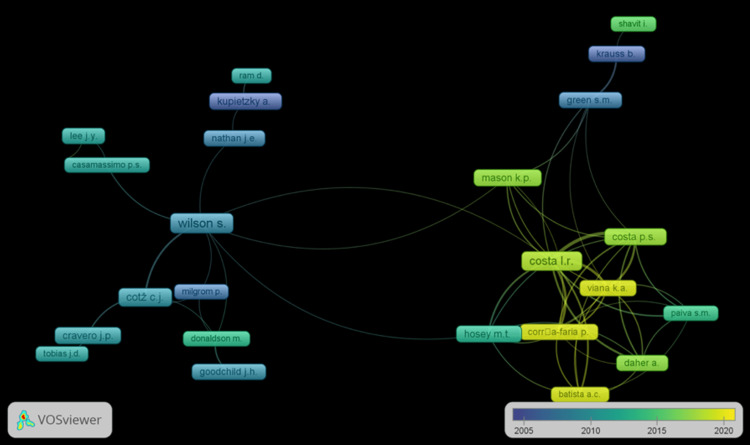
Author's collaboration

Authors, namely Wilson, Costa LR, and Mason, have inter-collaborations with authors from other countries. The collaboration mapping is elaborated in Figure 3.

Table 3 lists the top 10 cited articles. Pediatrics hosted two of the top 10 referenced papers. Two of the top 10 most quoted articles were written by Baruch Krauss [10,11].

**Table 3:** Top 10 cited articles on conscious sedation

Author	Citations	Year	Title	Journal
Henry R Costantino	434	2007	Intranasal delivery: physicochemical and therapeutic aspects [12]	International Journal of Pharmaceutics
CJ Cote	419	2002	Adverse sedation events in pediatrics: analysis of medications used for sedation [13]	Pediatrics
Baruch Krauss	374	2006	Procedural Sedation and Analgesia in Children [11]	Lancet
Baruch Krauss	332	2002	Sedation and analgesia for procedures in children [10]	New England Journal of Medicine
Joseph P cravers	285	2009	Incidence and nature of adverse events during pediatric sedation/anesthesia for procedures outside the operating room: report from the pediatric sedation research consortium [14]	Pediatrics
SP Nordt	210	1997	Midazolam: a review of therapeutic uses and Toxicity [15]	Journal of Emergency Medicine
M Mahmoud	181	2015	Dexmedetomidine: review, update, and future considerations of pediatric perioperative and periprocedural applications and limitations [16]	British Journal of Anaesthesia
American Society of Anesthesiologists	178	2018	Practice guidelines for moderate procedural sedation and analgesia 2018 [17]	Anesthesiology
Joel A Fein	171	2012	Relief of pain and anxiety in pediatric patients in emergency medical systems [18]	Pediatrics
Steven M Green	164	2004	Clinical Practice Guideline for emergency department ketamine dissociative sedation in Children [19]	Annals of Emergency Medicine

Eleven subject categories were used to distribute the 1064 articles. The top five subjects were medicine (46.9%), dentistry (38.6%), pharmacology (3.1%), biochemistry (1.8%), and neuroscience (1.5%), which denotes that conscious sedation research crosses disciplinary boundaries.

Of the 1064 articles, the majority (70%) were clinical studies, followed by reviews (17.4%), books (6.6%), conference papers (1.8%), letters (1.7%), editorials (0.9%), short surveys (0.7%) and notes (0.5%).

**Figure 4:**
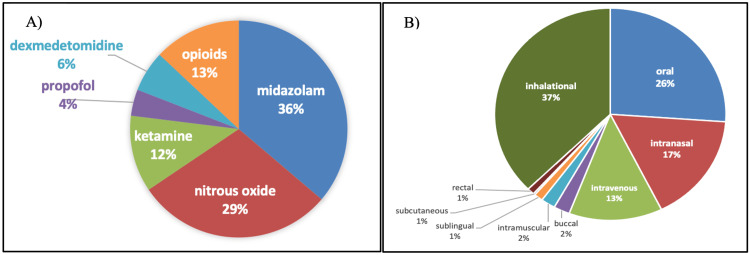
Topics addressed based on: A) the sedatives used; B) the routes of administration

Based on the study's primary objective, the topics covered were defined, including the choice of drugs and routes of administration (Figure 4). The most commonly used drug was midazolam, followed by nitrous oxide. The inhalational route is the most widely used, followed by the oral route. The publications on conscious sedation in special children and sedation in children with systemic conditions accounted only for 1%.

**Figure 5:**
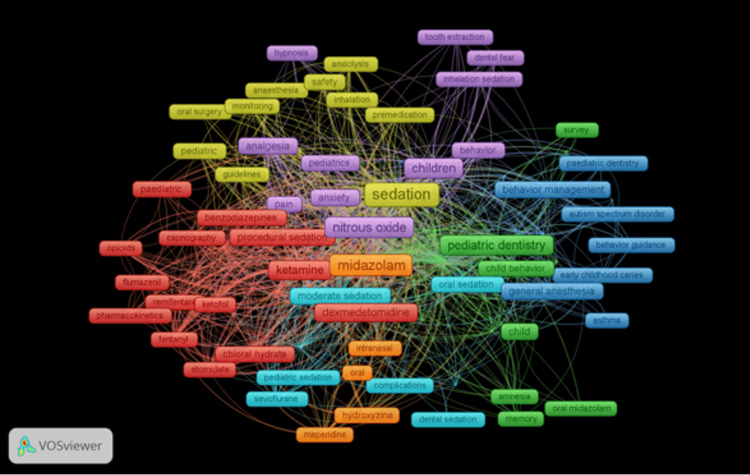
Density mapping of keywords

The 1064 articles contained 3320 keywords. Some of the top 10 keywords in Table 4 connected to the study's focus, such as "children." These included various sedatives, such as "midazolam," "nitrous oxide," "propofol," and "ketamine." Figure 5 displays a density visualization map of the top 50 terms. The top 10 keywords are listed in Table 4, along with their frequency and occurrences.

**Table 4:** Top 10 keywords

S. no.	Words	Occurrences	Frequency
1.	Sedation	157	182
2.	Conscious sedation	120	669
3.	Midazolam	120	179
4.	Nitrous oxide	89	252
5.	Pediatric Dentistry	68	386
6.	Children	62	213
7.	Ketamine	62	45
8.	Propofol	44	31
9.	Dentistry	40	424
10.	Dental Anxiety	35	49

Discussion

Decreasing the level of anxiety and pain in children undergoing dental procedures has been a challenge for pediatric dentists [20]. Clinicians regularly use sedation, but certain domains, like the ideal choice of drug and method of administration for children, remain ambiguous. Bibliometric analysis is an effective method for identifying global trends in the biomedical and therapeutic sectors [21]. This review used performance analysis and has identified 1064 total publications, 2433 contributing authors, and 10.7% productivity per year. A community's development, top authors, conceptual and intellectual maps, and trends have been evaluated using quantitative publication and citation data analysis.

As far as the authors know, this is the first bibliometric study to analyze the changing trends in conscious sedation in pediatric dentistry via the Scopus database without any journal restrictions. The most outstanding peer-reviewed publications, including books, journals, and conference proceedings, are found in Scopus. The journals in the Scopus database are audited annually to ensure they adhere to high criteria [22]. The author's connections, a list of their publications with citations, references, and the number of times each published work has been cited are all provided by Scopus as additional information about the writers. The Scopus database was therefore used in the current study to retrieve data.

"Conscious sedation," and "pediatric dentistry" were the search terms utilized, and a total of 2164 publications were published and indexed. One thousand sixty-four articles were included for bibliometric analysis after a preliminary search. For the top 10 keywords, a word frequency analysis was done, while "conscious sedation," "sedation," "pediatric dentistry," and "dentistry" were present in 99.15% (1055/1064) of the articles. This shows that the keyword-based search method effectively retrieves all pertinent articles. The use of keywords may provide insight into several aspects of the conscious sedation study. For instance, the sedatives utilized were geared toward the younger population. In the current investigation, age-related terms, including "children," "midazolam," and "nitrous oxide," were also identified as high-frequency keywords.

The variation in the volume of scholarly publications over time is a good gauge of the evolution of a discipline. Significant turning points may manifest as sudden shifts in the volume of publications. The current study discovered a two-decade period of modest literary increase. The number of publications was nearly three times higher in 2021 than in 2000, showing that the study of conscious sedation was in a phase of rapid advancement. One reason might be that the "guidelines for monitoring and management of pediatric patients before, during, and after sedation for diagnostic and therapeutic procedures" were revised in 2019 and published in the pediatric dentistry journal. However, the research may have reached a bottleneck because of a reduction in publications in 2009. With the emergence of a novel dexmedetomidine drug, publications started increasing in 2010. The regional distributions of scientific research production may represent differences in research capacities and technical advancements between various nations. Between 1996 and 2022, 75 nations or regions contributed to publications on conscious sedation, and 73% of these articles came from the top 10 countries, demonstrating that conscious sedation is a topic that is being researched globally. Seven of the top 10 nations were developed nations. Turkey, Brazil, and India were the three developing nations in the top 10 countries. Numerous explanations might exist. First, the nation's socioeconomic level and national oral health policies influence research capacity. Adequate medical supplies and staff trained to handle medical emergencies are necessary, especially for sedation. The core units of bibliometric analysis also include journals, authors, citations, and subject categories. The journal *Paediatric Dentistry* had the highest number of articles regarding conscious sedation. All the top 10 cited articles are reviews, updates, and guidelines. This indicates that evidence-based clinical decision-making has been increasingly emphasized while using conscious sedation. Citations demonstrate the sincere appreciation of work and the immediate, perceptible impact on international research. The current survey also lists the top citations received by nation, author, and publication, allowing scholars to concentrate on significant research and worldwide trends. The amount of publications an author publishes considerably impacts the h-index [23].

The study's design is an essential element that connects research to clinical practice. Most articles on conscious sedation were clinical studies. Clinical trials can mainly present the drug's effect on behavior changes while undergoing dental treatment, directly applied to regular clinical practice. Dentistry has recently placed a greater emphasis on evidence-based clinical decision-making. Therefore, greater emphasis should be given to well-designed, high-quality clinical studies that use novel and safe medications.

Concerning the topics addressed, most articles focused on the inhalational and oral routes of sedation. This represented that the drug acceptability by patients for the noninvasive-oral and inhalational routes is higher than the invasive parenteral routes. Focus on sedation for special children with autism, intellectual disability, down's syndrome, and cerebral palsy have been mentioned in the literature, yet the quantity of evidence is less.

Despite the limitation of choosing a single database, this study provides a relatively comprehensive view of scientific productivity related to conscious sedation in pediatric dentistry from 1996 to 2022.

Highlights of the Study

Language restrictions were removed from searches, potentially eliminating the bias of underestimating the research capacities of particular nations.

The authors have used both manual and software processes to give readers a comprehensive understanding of conscious sedation in pediatric dentistry.

## Conclusions

The findings of this review indicated a development in conscious sedation literature over the previous two decades. A lack of well-designed, high-quality clinical investigations persists involving novel drugs like dexmedetomidine. The number of publications evaluating adverse effects and their management, sedation for special children, is less compared to the publications assessing behavior changes using sedatives. These lacunae lay the groundwork for future research improvements in the field of conscious sedation.
